# Efflux pump inhibitor combined with ofloxacin decreases MRSA biofilm formation by regulating the gene expression of NorA and quorum sensing

**DOI:** 10.1039/d2ra06696c

**Published:** 2023-01-19

**Authors:** Xueer Lu, Guifeng Wang, Yunfeng Xie, Wenjian Tang, Biyong Liu, Jing Zhang

**Affiliations:** a Department of Clinical Laboratory, The Third People's Hospital of Hefei Hefei 230022 China; b Anhui Prevention and Treatment Center for Occupational Disease, Anhui No. 2 Provincial People's Hospital Hefei 230041 China lby770114@163.com hfzj2552@163.com; c School of Pharmacy, Anhui Medical University Hefei 230032 China

## Abstract

Carbonyl cyanide *p*-nitrophenylhydrazone (2e) displayed a lone or synergistic efficacy against MRSA (*RSC Adv.*, 2020, 10, 17854). In this work, the synergistic mechanism of 2e with ofloxacin was studied. MRSA2858 had potential for biofilm formation, and the value of MBEC of 2e alone was 0.78–1.56 μM, while that of 2e + ofloxacin was 0.39–0.78 μM. 2e combined with ofloxacin showed a synergistic anti-biofilm effect against MRSA. Efflux pump inhibitor 2e can better bind to NorA protein. After MRSA2858 was treated with 2e of 1/2MIC (0.78 μM) and ofloxacin of 1/8MIC (0.097 μM), the transcript levels of efflux genes (*norA*) and quorum-sensing (QS) regulatory genes (*agrA*, *sarA*, *icaA*, *hla*) were substantially down-regulated, and alpha-hemolysin (Hla) was inhibited by 99.15%. 2e combined with ofloxacin was more effective than 2e alone in reducing bacterial load *in vivo*. All in all, efflux pump inhibitor 2e enhanced the bactericidal activities of antibiotics through regulating the gene expression of NorA and QS system.

## Abstract 2

Carbonyl cyanide *p*-nitrophenylhydrazone (2e), an efflux pump inhibitor, displayed alone or synergistic efficacy against MRSA *in vitro* and *in vivo* through regulating the gene expression of NorA and QS system.

## Graphical abstract

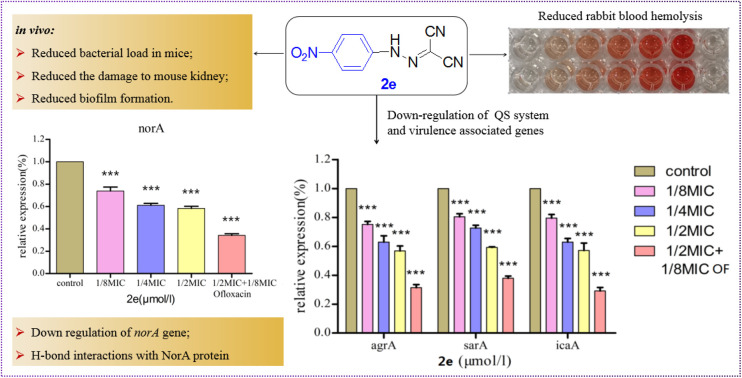

## Introduction

Methicillin-resistant *Staphylococcus aureus* (MRSA) has become a major global health threat.^1^ Biofilm-associated MRSA are difficult to eradicate, which can lead to acute to chronic infections, including diabetic foot infections, cystic fibrosis, and so on.^2–5^ Therefore, it is urgently needed to find unique therapeutic strategies or new chemical scaffolds against MRSA to counter this health challenge.

Carbonyl cyanide chlorophenylhydrazone (CCCP), an efflux pump inhibitor, is combined with antibiotics to enhance antimicrobial activity. CCCP reduces ATP production and increases membrane permeability in bacteria^6–9^ by interfering with the transmembrane electrochemical gradient and proton motive force.^6,10^ CCCP blocks the efflux of antibiotics outside the bacterial cells to decrease the biofilm-forming capacity of bacteria.^11^ However, it is toxic and has poor antibacterial activity when used alone.^12^ The efflux pumps also play a role in biofilm formation.^13^ The inhibition of efflux pumps helps to enhance the therapeutic efficacy of traditional antibiotics by affecting QS genes and virulence factors to control biofilm production.^11^

Recently, CCCP analog 2e was found to display alone or synergistic efficacy against MRSA.^13^2e could inhibit biofilm formation, effectively eradicated preformed biofilm, and showed low toxicity for human hepatic L02 cells.^14^ Studying the anti-biofilm activity of 2e and 2e combined with ofloxacin will help to understand the role and relationship of efflux pump and biofilm formation in MRSA.

Quorum sensing (QS) is one of the signaling mechanisms which directly aids in biofilm formation, and inhibiting QS can increase susceptibility to antibiotics. Formation and maintenance of biofilms are controlled by complex regulatory circuits such as QS and SaeRS two-component system (TCS).^15,16^ TCS is at the core of the QS system, which play an important role in bacterial survival, adaptation and pathogenesis, biofilm formation and virulence factor production.^17,18^ It is interesting how efflux pump inhibitors affect biofilm formation by QS and TCS.

When 2e was combined with ofloxacin, a synergistic anti-MRSA activity was observed (FICI = 0.28). In this work, we would further explore this synergistic antibacterial mechanism of efflux pump inhibitors on the biofilm formation by down-regulating QS system. The multi-drug resistant MRSA2858 strain was isolated and identified from clinical MRSA strains, which showed the potential of biofilm formation. 2e combined with ofloxacin inhibited biofilm formation of MRSA2858 by down-regulating the transcript levels of efflux gene (*norA*) and QS regulatory genes (*agrA*, *sarA*, *icaA*, *hla*), and decreasing alpha-hemolysin (Hla). This study provided a novel strategy for MRSA treatment.

## Materials and methods

### Reagents

**Fig. 1:**
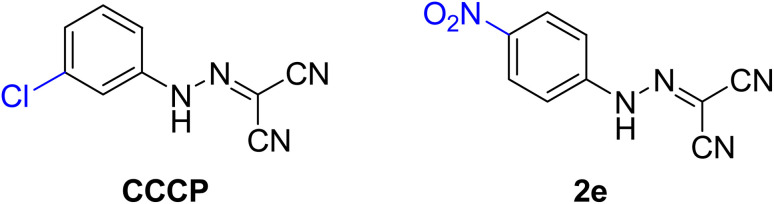
The chemical structures of CCCP and compound 2e.

All chemicals, reagents and solvents were purchased from commercial sources and used without further purification. Cation-adjusted Mueller–Hinton broth (CAMHB), crystal violet, trypsin–EDTA, tryptic soy broth (TSB), tryptic soy agar (TSA), phosphate-buffered saline (PBS), Dulbecco's modified Eagle's medium (DMEM), fetal bovine serum (FBS), and 96-well plates were all purchased from commercial vendors (BD, Cat.). CCCP and compound 2e was synthesized and their structures were shown in Fig. 1.^13^

### Strains and growth media

In this work, 16 clinical multi-resistant isolates of MRSA strains capable of establishing biofilms were collected. All of MRSA strains were resistant to three or more antimicrobial agents. *S. aureus* reference strain was obtained from American Type Culture Collection (ATCC 6538). The ability of the 16 strains to establish biofilms was tested using crystal violet (CV) assay (Table 2). MRSA2858 was the strongest biofilm-forming strain isolated from the patient's pus. Inoculums for the antimicrobial testing and the biofilm assays were prepared overnight in Mueller–Hinton (MH) broth and incubated at 37 °C.

**Table 1:** qRT-PCR primers in this study^23,24^

Primer	Sequence	Reference
*nor A*-F	GAGTGCTGGTATGGTAATGCC	Sivaranjani *et al.*, 2019
*nor A*-R	CCCTGGTCCTAAAATGAATCC	Sivaranjani *et al.*, 2019
*agr A*-F	TGCGAAGACGATCCAAAACA	Sivaranjani *et al.*, 2019
*agr A*-R	GGGCAATTTCCATAGGCTTTTC	Sivaranjani *et al.*, 2019
*sar A*-F	TGGTTCTCATCTCCCTTTGCTT	Sivaranjani *et al.*, 2019
*sar A*-R	GCGATGCTAATCTTCCTGGTG	Sivaranjani *et al.*, 2019
*ica A*-F	GGCTGGACTCATATTTGTAAGTTGG	Sivaranjani *et al.*, 2019
*ica A*-R	GTATTCCCTCTGTCTGGGCTTG	Sivaranjani *et al.*, 2019
*hla*-F	TTGGTGCAAATGTTTC	Sivaranjani *et al.*, 2019
*hla*-R	TCACTTTCCAGCCTACT	Sivaranjani *et al.*, 2019
*gyrB*-F	GGTGCTGGGCAAATACAAGT	Valliammai *et al.*, 2019
*gyrB*-R	TCCCACACTAAATGGTGCAA	Valliammai *et al.*, 2019

**Table 2:** MIC and biofilm formation values for the clinical isolates^a^

	Spec.	MIC of clinical used antibiotics^b^ (μg ml^−1^)																	MIC of 2e (μM)	Biofilm formation (OD595)^c^
		SAM	AMP	AMC	CRO	CLI	CIP	ERY	GEN	LVX	LNZ	OXA	PEN	RIF	QD	SXT	TCY	VAN		
MRSA0102	Sputum	≤8/4	>8	≤4/2	≤8	≤0.5	≤1	>4	≤4	≤1	≤4	>2	>8	≤1	≤0.5	≤0.5/9.5	≤4	2	1.562	0.599
MRSA0101	Sputum	≤8/4	>8	≤4/2	≤8	>4	≤1	>4	≤4	≤1	≤4	>2	>8	≤1	≤0.5	≤0.5/9.5	≤4	2	1.562	0.972
**MRSA2858**	**Pus**	**≤8/4**	**>8**	**≤4/2**	**32**	**>4**	**≤1**	**>4**	**>8**	**≤1**	**4**	**>2**	**>8**	**≤1**	**>2**	**≤0.5/9.5**	**≤4**	**2**	**1.562**	**1.511**
MRSA0612	Sputum	16/8	>8	>4/2	>32	≤0.5	>2	>4	>8	>4	≤1	>2	>8	>2	≤0.5	≤0.5/9.5	>8	≤0.25	3.125	1.461
MRSA10105	Blood	16/8	>8	≤4/2	≤8	≤0.5	≤1	≤0.5	≤4	≤1	2	>2	>8	2	≤0.5	≤0.5/9.5	8	1	1.562	0.366
MRSA05107	Blood	16/8	>8	>4/2	>32	>4	>2	>4	≤4	>4	2	>2	>8	≤1	≤0.5	≤0.5/9.5	≤4	2	3.125	0.445
MRSA1905	Sputum	≤8/4	>8	≤4/2	32	>4	≤1	>4	≤4	≤1	2	>2	>8	≤1	≤0.5	≤0.5/9.5	≤4	1	1.562	0.530
MRSA0352	Wound secretion	≤8/4	>8	≤4/2	≤8	≤0.5	≤1	>4	≤4	≤1	4	>2	>8	≤1	≤0.5	≤0.5/9.5	≤4	1	1.562	0.615
MRSA1257	Wound secretion	≤8/4	≤2	≤4/2	≤8	≤0.5	≤1	≤0.5	≤4	≤1	2	>2	0.12	≤1	≤0.5	≤0.5/9.5	≤4	1	1.562	0.404
MRSA1252	Wound secretion	16/8	>8	>4/2	>32	≤0.5	>2	≤0.5	≤4	≤1	2	>2	>8	≤1	≤0.5	≤0.5/9.5	≤4	2	3.125	1.313
MRSA0458	Wound secretion	≤8/4	>8	≤4/2	≤8	≤0.5	≤1	≤0.5	≤4	≤1	2	>2	>8	≤1	≤0.5	≤0.5/9.5	≤4	1	1.562	0.699
MRSA0961	Wound secretion	16/8	>8	>4/2	≤8	≤0.5	≤1	≤0.5	≤4	≤1	4	>2	>8	≤1	≤0.5	≤0.5/9.5	≤4	1	1.562	0.605
MRSA100902	Sputum	≤8/4	>8	≤4/2	≤8	≤0.5	≤1	≤0.5	≤4	≤1	≤1	>2	>8	≤1	≤0.5	≤0.5/9.5	≤4	1	1.562	0.468
MRSA101262	Wound secretion	≤8/4	>8	≤4/2	≤8	>4	≤1	>4	≤4	≤1	2	>2	>8	≤1	≤0.5	≤0.5/9.5	>8	2	3.125	1.031
MRSA2854	Pus	≤8/4	≤2	≤4/2	≤8	>4	≤1	>4	≤4	≤1	4	>2	2	≤1	≤0.5	≤0.5/9.5	≤4	2	1.562	0.416
MRSA1554	Pus	≤8/4	>8	≤4/2	≤8	>4	≤1	>4	≤4	≤1	2	>2	>8	≤1	≤0.5	≤0.5/9.5	≤4	1	1.562	0.203

a MRSA: methicillin-resistant *Staphylococcus aureus*.

b Antibiotics abbreviations: SAM: ampicillin/sulbactam, AMP: ampicillin, AMC: amoxicillin/clavulanic, CRO: ceftriaxone, CLI: clindamycin, CIP: ciprofloxacin, ERY: erythromycin, GEN: gentamicin, LVX: levofloxacin, LNZ: linezolid, OXA: oxacillin, PEN: penicillin, RIF: rifampin, QD: quinupristin/dalfopristin, SXT: trimethoprim/sulfa, TCY: tetracycline, VAN: vancomycin.

c Each data point represents a mean value of three independent experiment.

### MIC assay

The minimum inhibitory concentration (MIC) of tested compounds was determined using MH broth micro-dilution assay established by the Clinical Laboratory Standards Institute (CLSI) in 96-well microtest plates. Bacterial strains were grown overnight in MH broth at a concentration of 10^8^ CFU ml^−1^ in microtiter plates. Then, 100 μl of culture medium containing different concentrations of test compounds and reference antibiotics were added to the plates. The final bacterial concentration in the plate was 10^5^ CFU ml^−1^. After incubating for 20 h at 37 °C, the MIC was determined by observing the lowest concentration of tested compound which inhibited bacterial growth. All assays were performed in quadruplicate.

### Minimal biofilm eradication concentration (MBEC) determination

The antibiofilm activity of 2e was assessed *in vitro* against a mature biofilm formed in a flat-bottomed plate by an MRSA strain. For the assay, wells were filled with 200 μl of 1 : 100 dilution of a bacterial suspension cultured overnight in MH broth. After 24 h of incubation at 37 °C in static conditions, the medium was discarded, and planktonic cells were removed by thorough washing of the biofilm with sterile PBS. Then, 200 μl of MH broth supplemented with 2-fold dilutions of the compound (in the range 100–0.045 μM) were added in each well, and the plate was incubated further for 24 h at 37 °C.^19^ As an antibiotic control, dilutions of vancomycin, ofloxacin, rifampin and erythromicin in the range 100–0.045 μM were included. Biofilm formation was determined by crystal violet staining. To dissolve the crystal violet, 150 μl methanol per well was added to allow for minimal biofilm eradication concentration determination (MBEC) analysis.^20^ The optical density (OD) of each well was measured at 630 nm using an automated spectrophotometer (Bio-Tek Epoch-2, USA). Biofilm growth inhibition (%) = 100 − (OD_assay_/OD_positive_) × 100. Each experiment was repeated three times, with at least eight wells of each sample.

### Molecular docking and toxicity prediction

A structure based *in silico* procedure was applied to discover the binding modes of the active compounds to NorA protein active site. The CDOCKER of Discovery Studio Client v18.1.0 (DS) was conducted to explain SAR of series compounds and further guide the design of more effective and concrete NorA protein inhibitors. The ligand binding to the crystal structure of NorA protein with PDB ID: 3WDO was selected as template. The target enzyme was prepared with Prepare Protein of DS to ensure the integrity of target. The ligands (CCCP and 2e) were processed by full minimization of the small molecular in DS. Then title compounds were docked into the active site of protein using CDOCKER. The view results of molecular docking were extracted after the program running end, each docking result was analyzed for interaction and their different pose. Those poses with the lowest –CDOCKER_INTERACTION_ENERGY values were regarded as the most stable and picked to analysis binding interactions with target enzyme visualized.

### Molecular dynamics simulations

The molecular dynamics (MD) simulations were performed in Yinfo Cloud Computing Platform using AmberTools 20 package with AMBER ff19SB and GAFF force fields for NorA and compound 2e, respectively.^21,22^ The system was solvated by a truncated octahedron (or cubic) water box using OPC water model with a margin of 10 Å, the net charge was neutralized by (0.15 M of NaCl) sodium ions. Periodic boundary condition (PBC) was used and the net charge neutralized by Na^+^ (or Cl^−^) ions (or 0.15 M of NaCl). Nonbonded van der Waals interactions were calculated using the Lennard-Jones 12–6 potentials with a 10 Å cutoff, while long-range electrostatics were treated using the Particle Mesh Ewald (PME) algorithm. The SHAKE algorithm was applied to constrain bonds involving hydrogen atoms. To removed improper atom contacts, the system was first minimized by (1) the 5000 steps of steepest descent and the 5000 steps of the conjugate gradient, under a harmonic constraint of 10.0 kcal (mol Å^2^)^−1^ on heavy atoms; (2) relaxing the entire system by steepest descent and conjugate gradient each in 20 000 steps. And then the system was gradually heated up to 300 K by a 20 ps NVT simulation. The equilibration phase was carried out in two steps: (1) a 200 ps NPT simulation with constraints on heavy atoms followed by (2) a 500 ps NVT simulation without restraint. The temperature and pressure were maintained at 300 K and 1 atm using the Berendsen thermostat and Monte Carlo barostat, with coupling constant and relaxation time of 1 ps. Finally, the system was subjected to a 20 ns NPT simulation with a time step of 1 fs. The root-mean-square deviation (RMSD), root-mean-square fluctuation (RMSF), and hydrogen bonds were analyzed by the Cpptraj module.

MM/PB(GB)SA calculations. The binding free energies were calculated using the Molecular Mechanics Poisson–Boltzmann Surface Area (MM/PBSA) method implemented in AmberTools 20 for 200 snapshots from the MD trajectory. For each snapshot, the free energy was calculated for protein NorA, compound 2e, and the complex NorA–2e using a single trajectory approach. The total binding free energy was calculated according to the following equation:Δ*G*_bind_ = Δ*G*_complex_ − (Δ*G*_receptor_ − Δ*G*_ligand_)Δ*G*_bind_ = Δ*E*_MM_ + Δ*G*_solv_ − *T*Δ*S*Δ*E*_MM_ = Δ*E*_vdw_ + Δ*E*_ele_Δ*G*_solv_ = Δ*G*_PB_ + Δ*G*_SA_where Δ*E*_MM_ denotes the gas-phase interaction energy between the receptor and the ligand (including van der Waals energy contribution (Δ*E*_vdw_) and electrostatic energy contribution (Δ*E*_ele_)); Δ*G*_PB_ and Δ*G*_SA_ are the polar and nonpolar components of the de-solvation free energy, respectively; *T*Δ*S* represents the conformational entropy contribution at temperature *T*. Here, Δ*G*_PB_ was determined by the Poisson–Boltzmann approximation model, while Δ*G*_SA_ was estimated based on the solvent accessible surface area model by the method: Δ*G*_SA_ = *γ* × SASA + *β*, where the values of the constants *γ* and *β* were 0.00542 kcal Å^−2^ and 0.92 kcal mol^−1^, respectively. The solvent probe radius and ionic strength were set to be 1.4 Å and 0.15 mM, respectively. The interior and exterior dielectric constant of MM/PBSA calculation systems was 1.0 and 80.0.

### Effect of 2e on gene expression of MRSA strains

Total RNA from MRSA cultures treated with 2e alone or in combination with ofloxacin for 24 h was extracted using the TRIzol method for RNA extraction and converted to cDNA using SuperScript™ III First-Strand Synthesis SuperMix for qRT-PCR (Invitrogen, USA). The PCRs were performed in a 20 μl volume and contained Roche SYBR Green PCR Master Mix. The fold change in gene expression was calculated by the 2^−ΔΔ*c*_t_^ method with *gyrB* as the housekeeping gene. Specific primers are listed in Table 1. Amplification was performed in a gradient thermal cycler (Bio-Rad, Hercules, USA). All samples were analyzed in triplicate.

### Hemolytic activity

Hemolytic activity can be assessed by a rabbit red blood cell lysis technique, which consists of bringing the red blood cells into contact with the MRSA2858 or 2e, and quantifying the hemolytic activity of alpha-hemolysin (Hla) by spectrophotometric determination. Samples (100 μl) of MRSA2858 culture (1 × 10^9^ CFU ml^−1^) were filtered and were added to 900 μl hemolysin buffer (0.145 mol per l NaCl, 0.02 mol per l CaCl_2_) and 25 μl of defibrinated rabbit blood. Rabbit blood was washed three times with 0.9% NaCl and resuspended to a concentration of 0.5 × 10^8^ cells per ml, as determined by manual cell count. The solution was incubated for 15 min at 37 °C. Unlysed blood cells were pelleted by centrifugation (5500*g*, 1 min). The hemolytic activity of the supernatant was determined by measuring the optical density at OD_541._ Sterile culture medium served as the standard for 0% hemolysis, and a bacterial culture supernatant devoid of any inhibitor (control) was designated as the standard for 100% hemolysis. The percentage of hemolysis inhibition was calculated by comparison with the control culture. All assays were performed in triplicate.

### *In vivo* anti-bacterial activity

SPF female BALB/c mice were selected to avoid the re-infection induced by the fighting and barbering of males. SPF female BALB/c mice (aged 6–8 weeks; 18–21 g) were purchased from Changzhou Cavens Experimental Animal Co., Ltd, Animal certificate number: SCXK (SU) 2016-0010; mice were maintained with SPF food and water for 1 week, 6 mice per group. The test strain was refreshed on MH broth at 37 °C for 24 h. From the overnight culture preparation, a single colony was diluted into 5 ml of MH broth and incubated overnight at 37 °C. A log-phase subculture was obtained by adding 150 μl of overnight subculture in 10 ml MH broth and incubated for a further 2–3 h. A full dilution of the bacterial cell suspension in saline was achieved by washing (3220 g, 10 min) and the OD_600_ in saline determined. The suspension of 1.5 × 10^9^ CFU ml^−1^ solution was diluted in MH broth into the final bacterial concentration of 10^5^ CFU ml^−1^. Mice were infected by intraperitoneal administration of MRSA2858 in 0.2 ml of MH broth.

After bacterial challenge for 2 h, mice were randomized to receive subcutaneous injection of saline as a control, ofloxacin (5 mg kg^−1^), 2e (5 mg kg^−1^), or 2e combined with ofloxacin group (5 mg kg^−1^ + 5 mg kg^−1^). To assess bacterial clearance, six mice in each group were euthanized, and bacterial counts were determined in the kidney and blood from each animal after bacterial challenge for 24 h. In the MRSA infection model, parts of kidney tissues were fixed in 10% neutral buffered formalin for 24 h, and then their morphologies were observed using H&E staining.

### Statistic analysis

Statistical analysis was performed using SPSS 13.0 software package, and one-way ANOVA followed was used to assess the significance. A *p*-value ≤ 0.05 was set as statistically significant. Data were analyzed by Microsoft Excel 2003 spreadsheet software, and expressed as the mean ± standard deviation.

## Results

### Antibacterial activity of 2e

The results showed that 2e performed the best against *S. aureus* with MIC value of 1.56 μM, and against MRSA with MIC value of 1.56 μM, which was superior to the front-line antibiotics including cefoxitin and linazolid.^14^ As shown in Table 2, 16 clinical MRSA were isolated from different specimens, and all were resistant to at least three antimicrobial agents of furantoin, penicillin, rifampicin, tetracycline, tegacyclin, ciprofloxacin, erythromycin, gentamicin, linezolid, benzacillin or clindamycin. Among them, MRSA2858 was a clinical isolate with multi-drug resistance and the strongest biofilm formation ability. Although certain antibiotics have MIC values even more favorable than compound 2e in Table 2, levofloxacin and ciprofloxacin are alternative or complementary drugs and are not clinical first-line anti-MRSA agents according to CLSI guidelines.

Studying the synergistic mechanism of 2e with ofloxacin will help to enhance the therapeutic efficacy of traditional antibiotics against resistant bacteria. Data indicated that 2e strongly inhibited the bacterial growth of all clinical strains with the potency of MIC values less than 1.56 μM and the results had no statistically significant differences for all strains, which validated 2e as a promising anti-MRSA agent.

### Anti-biofilm activity of 2e

To assess the anti-biofilm potential of 2e, the MRSA clinical isolates were assayed *in vitro* in a preliminary set of experiments for their ability to form adherent biofilm layers under static conditions.^19^ Clinical strain MRSA2858 demonstrated high biofilm capacity and planktonic cells that were susceptible to 2e (Table 2). MRSA2858 biofilms were initially grown for 24 h and then treated with dilutions of 2e and vancomycin, ofloxacin, rifampin, erythromicin, as antibiotic control, for a further 24 h. Biofilm production and eradication were quantitatively evaluated using crystal violet staining.

**Fig. 2:**
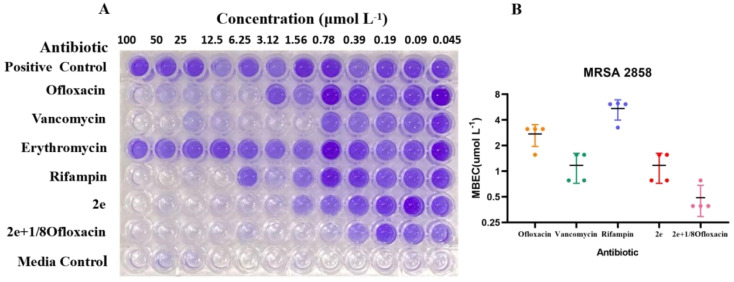
Anti-biofilm activity of 2e alone or synergistic efficacy against MRSA. MBEC assessment using crystal violet staining (A) to assess the amount of MRSA2858 biofilm remaining (B) after 24 h incubation with 2e, vancomycin, ofloxacin, rifampin and 2e + 1/8ofloxacin (biofilm initially established by 48 h growth in TSB). Erythromycin resistance is shown in (A). Data are *n* = 4 biologically independent samples.

As shown in Fig. 2 and Table 3, 2e alone and combination with 1/8MIC ofloxacin was active against MRSA biofilms, the value of MBEC was 0.78–1.56 μM and 0.39–0.78 μM, similar to it MIC, superior to those of vancomycin, ofloxacin, rifampin and erythromycin (Fig. 2A). Although low level of β-lactam antibiotic was reported to induce biofilm formation of *S. aureus*,^25,26^2e combined with subinhibitory concentration of ofloxacin (1/8MIC) showed a synergistic anti-biofilm effect against MRSA (Fig. 2B).

**Table 3:** Anti-biofilm effects of compounds on MRSA2858

Compound	Ofloxacin	Vancomycin	Rifampin	2e	2e + 1/8ofloxacin
MBEC (μM)	2.73 ± 0.78	1.17 ± 0.45	5.43 ± 1.46	1.17 ± 0.45	0.48 ± 0.20

### Molecular docking and toxicity prediction

**Fig. 3:**
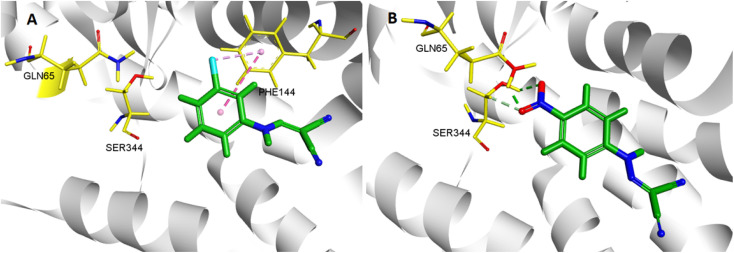
3D interaction patterns CCCP (A) and 2e (B) with NorA (PDB: 3WDO) using DS client v19.1.0 analysis.

In the molecular docking study, two ligands (CCCP and 2e) were docked into NorA protein, and they were located in the same activity site. 4-NO_2_ of 2e formed hydrogen bond interaction with Gln 65 and Ser 344, while CCCP cannot interact with these two amino acid residues. Therefore, 2e can better bind to NorA protein. Structure–activity relationship (SAR) analysis showed that 4-NO_2_ made 2e adopt a different binding method into the NorA protein (Fig. 3), which implied that 2e may be a new NorA protein inhibitor.

In order to assess the feasibility of 2e*in vivo* application, we performed an *in silico* analysis of physicochemical, pharmacokinetic and toxicological properties using Discovery Studio2018 software. Although 2e was predicted to be carcinogenic risk and mutagenic risk in humans, this cannot be considered a major problem in a drug discovery process, as many widely used drugs in the market are exist these risks. Outstandingly, the chronic oral lowest toxic dose of 2e (90.2 mg kg^−1^) is much higher than the actual dose (5 mg kg^−1^).

### Molecular dynamics simulation

**Fig. 4:**
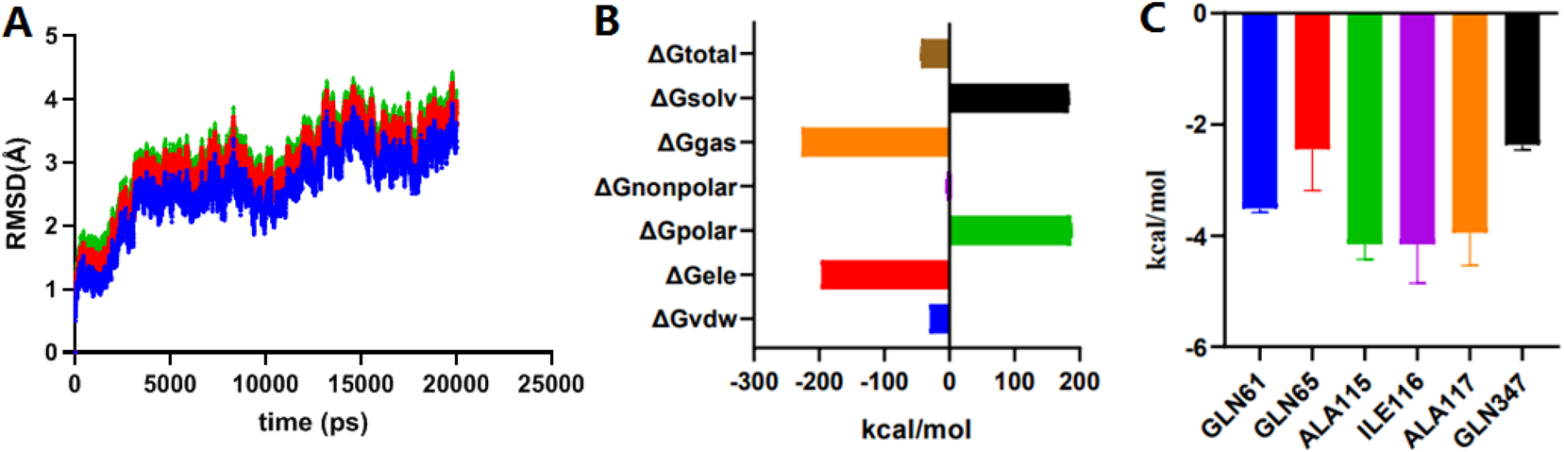
The RMSD plots of complex NorA–2e*vs.* time (A); total binding free energy and its component (B); residue contribution for receptor–ligand combination (C).

MD simulation mainly relies on Newtonian mechanics to simulate the motion of a molecular system. This approach can not only obtain the trajectory of atoms but also enables the observation of various microscopic details during atomic motion. Compound 2e was docked into the NorA protein pocket and then subjected to MD simulation to examine the structural stability of simulation system. MD simulation of complexes was performed for 20 ns period. In this study, the kinetic energy of 2e started to increase by temperature and time until a steady state was reached. As shown in Fig. 4A, before 10 000 ps, the entire system of the complex NorA–2e was in a stable state. After 10 000 ps, an obvious upward trend was emerged, especially at 12 000 ps, after which the overall system was relatively stable, and the wave range of RMSD was within 1 Å. Subsequently, calculated by the MM-PB(GB)SA method (Fig. 4B), the total binding free energy was −43.76 kcal mol^−1^ for the complex of NorA–2e, and the van der Waals energy was −30.01 kcal mol^−1^. Fig. 4C was the contributions of hot residues in the binding pocket of NorA protein. Under normal circumstances, a residue with lower interaction energy than −1 kcal mol^−1^ was considered to be essential for ligand recognition and combination. As disclosed in docking studies, hydrogen bonding of 2e with Gln65 (−2.44 kcal mol^−1^) was significant for binding to NorA protein.

### Effect of 2e on *norA* gene expression

**Fig. 5:**
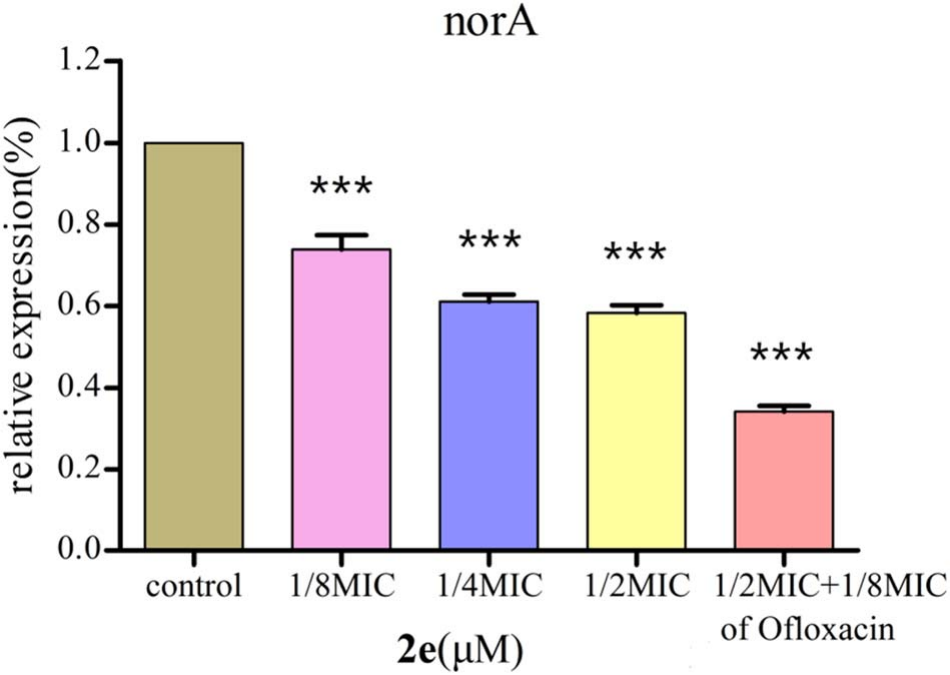
Effect of 2e and 1/8MIC ofloxacin (0.097 μM) on *norA* genes expression in MRSA. The control was the untreated biofilm control group (***p < 0.01).

2e was used to examine the effect on the expression of *norA* gene in MRSA2858 using qRT-PCR (Fig. 5). 2e alone significantly down-regulated the gene expression of *norA* by 41.8%. Combination of 2e with 1/8MIC ofloxacin reduced the expression of *norA* by 65.9%. The results showed that 2e combined with low concentration of ofloxacin showed a synergistic inhibitory effect against MRSA by regulating NorA efflux pump.

### Effect on expression of QS regulatory genes

**Fig. 6:**
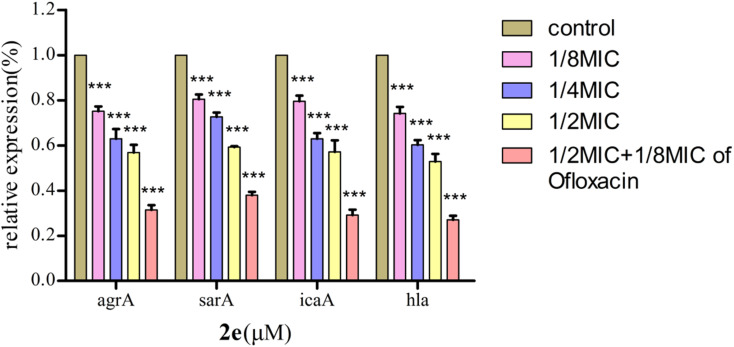
Effect of 2e on QS regulatory gene expression in MRSA2858 (***p < 0.01).

The QS system plays a role in the precise regulation of genes controlling virulence factors and biofilm formation in MRSA. 2e exerted dose-dependent inhibitory effects on virulence phenotypes (*hla*) regulated by QS in MRSA using qRT-PCR (Fig. 6). Moreover, the expression levels of QS regulatory genes, including *agrA*, *sarA* and *icaA*, were repressed after 2e combined with 1/8MIC ofloxacin treatment in a dose-dependent manner, and genes *hla*, *agrA*, *sarA* and *icaA* were down-regulated by 73.0%, 68.7%, 62.1% and 71.0%, respectively. These results confirmed the expression of QS regulatory genes (*agrA*, *sarA*, *icaA*, *hla*) were substantially down-regulated in treated with 1/2MIC 2e at 0.78 μM and 1/8MIC ofloxacin at 0.097 μM.

### Effect on MRSA2858 virulence

**Fig. 7:**
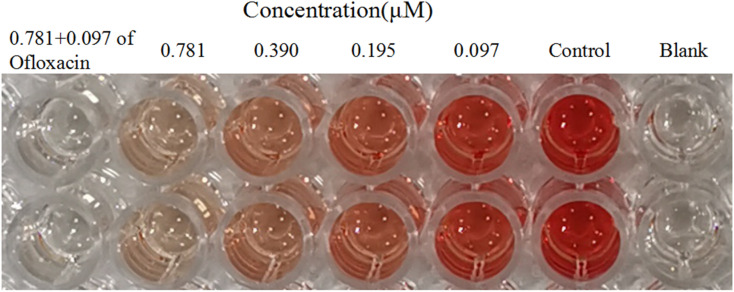
Inhibitory effect of 2e on rabbit blood hemolysis by MRSA2858.

Alpha-hemolysin (Hla) was a major virulence factor in the pathogenesis of *S. aureus* infection, being active against a wide range of host cells. Hla created holes in the membranes of various host cells, such as immune system cells and erythrocytes. The amount of hemoglobin released from lysed red blood cells was taken as a measure of Hla production. 2e significantly decreased the level of hemolysis in a dose-dependent manner at 0.097–0.781 μM. When 2e was combined with 1/8MIC ofloxacin, the inhibition rate of α-hemolysin was 99.15%, while the inhibition rate of α-hemolysin for 2e alone was 93.75% (Table 4, Fig. 7). These results suggested that the synergistic antibacterial effect came along with the virulence inhibition of MRSA.

**Table 4:** Inhibitory effect of 2e on rabbit blood hemolysis by MRSA2858

Compound (μM)	% inhibition of hemolysis of rabbit blood at				
	0.781 + 0.097 of ofloxacin	0.781	0.390	0.195	0.097
2e	99.15 ± 0.64	93.75 ± 1.50	83.85 ± 2.24	68.88 ± 0.74	43.73 ± 0.91

### *In vivo* antibacterial activity

#### Effect of 2e or/and ofloxacin treatment on bacterial load of blood and kidney

**Fig. 8:**
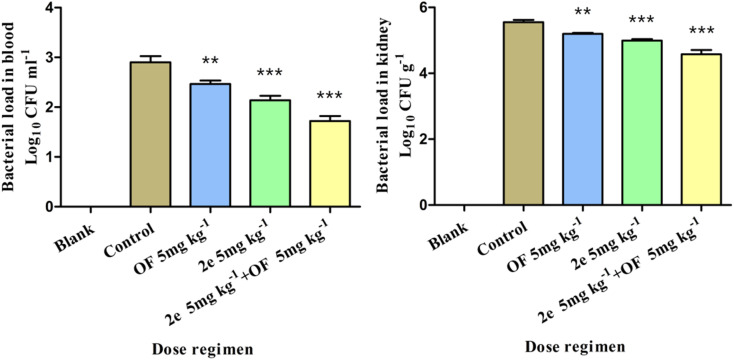
Effect of treatment with 2e or/and ofloxacin on bacterial load of different tissues (comparison between each administration group and control group, **p < 0.05, ***p < 0.01).

The antibacterial effect of 2e on MRSA was evaluated in the mouse abdominal infection model. MRSA2858 was used to observe the effect of 2e, ofloxacin, and their combination on reducing the bacterial load in the blood and kidney of mice. 2e combined with ofloxacin could significantly reduce the bacterial load in the blood and kidney of mice, bacterial colonies counts were dropped to 1.18 and 0.97 log_10_ CFU ml^−1^, respectively (Fig. 8). The efficacy of 2e combined with ofloxacin was superior to that of 2e or ofloxacin, which suggested that 2e combined with ofloxacin could effectively reduce the MRSA abdominal infection.

#### Effect of 2e or/and ofloxacin treatment on hematological parameters *in vivo*

Hematological studies provided a good indication of the progress of bacterial infection as well as treatment. WBC, neutrophils and monocytes may drop abruptly due to massive consumption in a short period of time by acute bacterial infection. MRSA was known to contain hemolysin that can help in targeted killing of host cells. A reduction in Hb and RBC was noticed in untreated infected group. In treated group, all compounds displayed improvement in haemalogical parameters. After treatment with 2e combined with ofloxacin, the best adjustment of RBC, WBC and monocytes was observed, and HGB and neutrophil count were similar to negative control, showing their higher potency in controlling infection (Table 5).

**Table 5:** Effect of 2e or/and ofloxacin treatment on hematological parameters of mice at 24 h post infection

Blood parameter	Groups				
	Negative control	Positive control	2e (5 mg kg^−1^)	OF (5 mg kg^−1^)	2e (5 mg kg^−1^) + OF (5 mg kg^−1^)
WBC (10^9^ l^−1^)	10.48 ± 0.83	4.64 ± 1.12^b^	5.94 ± 0.51^b^	4.75 ± 1.11^b^	8.52 ± 0.44^a^
NE (10^9^ l^−1^)	1.98 ± 0.39	0.98 ± 0.33^a^	1.25 ± 0.21^a^	1.15 ± 0.29^a^	1.60 ± 0.51
MONO (10^9^ l^−1^)	8.38 ± 0.46	3.00 ± 0.91^b^	3.66 ± 0.39^b^	4.21 ± 1.26^b^	5.71 ± 1.16^a^
RBC (10^12^ l^−1^)	11.26 ± 1.35	8.98 ± 0.42^a^	9.70 ± 0.24	9.59 ± 0.23	9.81 ± 1.19
HGB (g l^−1^)	168.67 ± 18.01	135.33 ± 6.51^a^	145.67 ± 2.08	143.00 ± 1.00	152.00 ± 14.73

a *p* < 0.05.

b *p* < 0.01, compared with negative group. Data are presented in term of mean ± SD.

#### Effect of 2e or/and ofloxacin on the pathological changes of kidney tissue

**Fig. 9:**
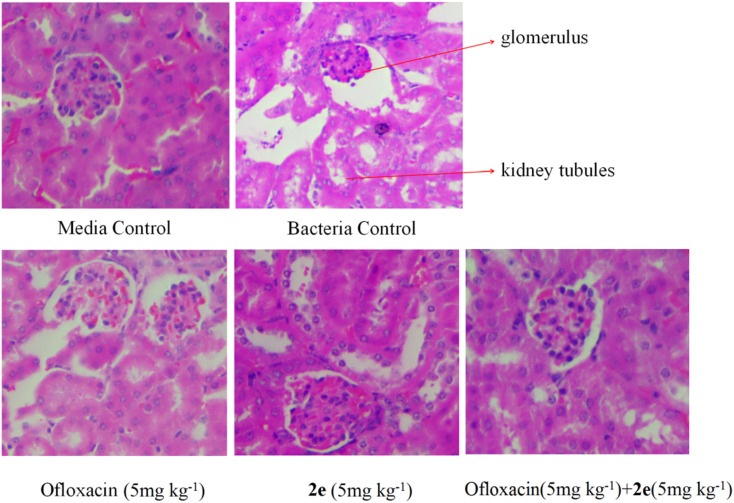
Effect of 2e or/and ofloxacin on the pathological changes of kidney tissue caused by MRSA. The kidney was stained with H&E for histological examination.

Firstly, kidney tissues were removed and fixed in 10% formaldehyde. Secondly, after dehydration in gradient concentration of alcohol, the tissues were embedded in paraffin and sliced. Finally, sections were stained by H&E, and imaged under an optical microscope. As shown in Fig. 9, in the blank control group, normal morphology of the glomeruli and proximal tubules were clearly shown in the kidneys of mice, while in abdominal infection mouse model, the kidneys were severely damaged, resulting in renal tubular epithelial injury, inflammatory cell infiltration, renal tubular cell swelling, and tubular dilatation. Therefore, the treatment of 2e combined with ofloxacin significantly improved the necrosis of kidney tissue and inflammatory infiltrating cells caused by MRSA in model mice.

## Results and discussion

MRSA infection is a serious threat to human health. The ability of biofilm formation in MRSA can lead to resistance to most currently used antibiotics.^27–29^ Novel antibacterial agents can be developed to inhibit their function, to help reduce the virulence of bacteria and avoid drug resistance. There's different methods for evaluating biofilm formation, and the most common ones are CV staining for biomass and XTT (tetrazolium salt reduction) assay for viability.^30^ Because CV staining is often used for quantification of *S. aureus* biofilm formation,^31^ the CV method is more suitable to evaluate the inhibition of 2e for biofilm formation and biomass production.

This study showed that 2e could inhibit the biofilm formation of MRSA *in vitro*. The transcription profiling demonstrated that the NorA efflux pathway was obviously inhibited by 2e treatment. As one of the most important efflux pump genes of *S. aureus*, *norA* gene was over expressed in 43% of strains.^32,33^ NorA was capable of extruding multiple structurally dissimilar substrates such as hydrophilic fluoroquinolones.^34^ The use of efflux pump inhibitors (EPIs) could decrease the MIC of antibacterial agents, increase susceptibility restoration in clinical resistant strains, and inhibit biofilm formation. NorA EPIs were capable of restoring drug susceptibility in resistant strains have been found, such as reserpine, verapamil, omeprazole, chlorpromazine, *etc.*^35^ The efflux pump can expel metabolites, toxic substances and antibacterial drugs out of the membrane of bacteria, which helps to maintain the vitality and virulence of starving bacteria in the biofilm. Therefore, the expression level of efflux pump was positively correlated with the formation of biofilm.^34^ We found that 2e markedly inhibited NorA pump activity.

The norA pump is regulated by the QS system. QS system has been known to be essential for robust biofilm production and development in numerous bacteria, including clinical multi-drug resistant isolates.^36^ Consistent with these observations, 2e treatment could down-regulate the expression of QS regulatory genes (*agrA*, *sarA*, *icaA*, *hla*) in a dose-dependent manner, decrease the virulence by blocking the NorA efflux pathway, and contribute to the biofilm inhibitory activity. Although biofilm development in MRSA is *ica* independent,^37^ we observed that the regulatory gene of MRSA2858 was *ica* dependent.

One of the key features of *S. aureus* infection was the production of a series of virulence factors, including secreted enzymes and toxins.^38^ In this study, the pore-forming toxin alpha-hemolysin (Hla) was a *S. aureus* secreted factor which participated in the activation of the NorA efflux pathway. This study showed that the anti-virulence activity of compound 2e combined with ofloxacin was superior to that of 2e alone against MRSA.

QS-controlled biofilm formation and virulence factor secretion by MRSA in clinical settings has remained controversial due to emerging drug resistance. Therefore, it is important to find diverse molecules for anti-biofilm or anti-QS activities.

## Conclusion

It was urgent for the discovery of new anti-resistant agents with anti-biofilm and anti-efflux pump activity, especially therapeutic alternatives for multi-drug resistant MRSA. Carbonyl cyanide *p*-nitrophenylhydrazone 2e alone or combined with ofloxacin was found to have potent anti-MRSA activity. 2e combined with ofloxacin was exhibited anti-resistant activity through inhibiting NorA efflux pump, as well as decreasing biofilm formation, decreasing Hla and regulating QS systems of MRSA. Therefore, our results are significant and relevant to find novel therapeutic methods against MRSA to reduce the use of antibiotics.

## Ethical statement

In this study, MRSA strains were isolated and identified from diverse sources including blood, peritoneal fluid, and purulent leukorrhea of clinical infected patients, and the samples were collected by technically trained persons. These samples were evaluated and permitted by the Institutional Ethical Committee (IEC), Anhui No. 2 Provincial People's Hospital, China (IEC No. 2019814). The procedures for maintenance and treatment of laboratory animals were approved by the Animal Care and Use Committee of the Anhui No. 2 Provincial People's Hospital. All experiments were performed in accordance with the National Institutes of Health Guide for the Care and Use of Laboratory Animals.

## Author contributions

Xueer Lu, Guifeng Wang and Yunfeng Xie contributed to the conception of the study and performed the experiment; Xueer Lu and Guifeng Wang contributed to analysis and manuscript preparation; Xueer Lu and Wenjian Tang helped perform the analysis with constructive discussions. Biyong Liu and Jing Zhang performed the data analyses and wrote the manuscript. All authors listed have made a substantial, direct, and intellectual contribution to the work and approved it for publication.

## Conflicts of interest

The authors confirm that this article content has no conflict of interest.

## Supplementary Material
